# SLEEP QUALITY IN LATER LIFE: HOW DOES IT AFFECT RISKY DRIVING BEHAVIORS AND CRASH INCIDENCE?

**DOI:** 10.1093/geroni/igae098.2575

**Published:** 2024-12-31

**Authors:** Anne Barrett, Brianna Soulie, Hope Mimbs

**Affiliations:** Florida State University, Tallahassee, Florida, United States; Florida State University, Tallahassee, Florida, United States; Florida State University, Tallahassee, Florida, United States

## Abstract

Among the leading causes of motor vehicle injury and fatality is drowsy driving. Most studies, however, focus on young drivers or those in shift positions (e.g., truck drivers). Little is known about how sleep quality affects the driving behavior and injury risk of older adults. Their risk may be heightened by the increased susceptibility to sleep disturbances that can occur in later life. Using data from a survey of Floridians aged 50 and older conducted between December 2020 and April 2021(n=4,199), we examined the how self-rated sleep quality and sleepiness while driving are associated with two driving outcomes: frequency of engaging in risky driving behavior (e.g., speeding, texting/emailing) and having experienced a crash or near-crash in the past year. Results of multivariate regression models revealed that reporting sleepiness while driving and lower sleep quality predicted greater likelihood of having experienced a crash or near-crash. Reporting lower sleep quality also predicted engaging more frequently in risky driving behavior. Other significant predictors of risky driving behavior or likelihood of a crash or near-crash included gender, socioeconomic status, marital or partnership status, pain frequency, self-reported hearing ability, self-reported frequency of memory problems, depressive symptoms, driving frequency, self-rated driving ability, and rural residence. Our study highlights the importance of examining how change in sleep patterns over the life course may affect driving behaviors and roadway safety.

